# Editorial: Recent advances in pheochromocytoma and paraganglioma: molecular pathogenesis, clinical impacts, and therapeutic perspective, volume II

**DOI:** 10.3389/fendo.2025.1605189

**Published:** 2025-05-02

**Authors:** Ichiro Abe, Farhadul Islam, Suja Pillai

**Affiliations:** ^1^ Department of Endocrinology and Diabetes Mellitus, Fukuoka University Chikushi Hospital, Chikushino, Fukuoka, Japan; ^2^ Department of Biochemistry and Molecular Biology, University of Rajshahi, Rajshahi, Bangladesh; ^3^ School of Biomedical Sciences, Faculty of Medicine, University of Queensland, Saint Lucia, QLD, Australia

**Keywords:** pheochromocytoma, paraganglioma, molecular pathogenesis, clinical impacts, therapeutic perspective

This Research Topic encompasses current perspectives on the molecular mechanisms, genetics, clinical manifestations, and novel therapeutic management of Pheochromocytomas and Paragangliomas (PPGLs). In the previous Research Topic, Recent Advances in Pheochromocytoma and Paraganglioma: Molecular Pathogenesis, Clinical Impacts, and Therapeutic Perspective, *i.e.* knowledge of the molecular and genetic spectrum, mechanisms of complications, and novel therapeutic options of PPGLs were illustrated. The knowledge of PPGLs has been advancing; thus, in this Research Topic, we have updated the current understanding of PPGLs.

PPGLs are relatively rare neuroendocrine tumors derived from chromaffin cells in the adrenal medulla and/or autonomic nervous system ganglia. Their clinical importance, including various associated complications, is due to catecholamine excess (1, 2). Moreover, PPGLs could lead to pheochromocytoma multisystem crisis (PMC), which is a life-threatening endocrine emergency with reported mortality as high as 85-90% (3). Thus, updated knowledge of management of PPGLs, especially based on the molecular mechanisms and genetics, is necessary. Meanwhile, according to the WHO’s classification in 2017, all pheochromocytomas could have metastatic potential and no histological system to assess the biological aggressiveness. Hence, “Malignant pheochromocytoma” in the 2004 WHO classification was replaced with “Metastatic pheochromocytoma” in the 2017 WHO classification (4). Considering the above, an updated understanding of clinicopathological advances and the management of PPGLs, mainly based on molecular mechanisms and genetics, is necessary (2, 4–7).

In this Research Topic, Saavedra et al. reviewed the clinical presentation, management, and treatment of patients with PPGLs. In this review article, early diagnosis, combined with an understanding of the genetic landscapes and comprehensive treatment strategies, was described as necessary to improve outcomes for patients with PPGLs. Nevertheless, surgery is the mainstay of treatment for patients with PPGLs. The utilization of Da Vinci robot-assisted laparoscopic surgery contributed to a favorable prognosis for a patient. In addition, Yang et al. reported a case of paraganglioma with a newly detected *EPAS1* mutation, which may be the primary driver of the disease.

Some therapeutic options in patients who could not receive surgery and/or have metastatic PPGLs have been reported in this Research Topic. For example, Cyclophosphamide-Vincristine-Dacarbazine (CVD) chemotherapy is a conventional therapeutic option and was reported to be the first-line treatment for PPGLs with *SDHB*-mutation previously (8). Zhang et al. performed CVD chemotherapy for a patient with metastatic paraganglioma having *SDHB*-mutation and treated it very effectively.

Recently, radiotherapy for metastatic PPGLs has evolved. Gubbi et al. performed a phase 2 trial of [^177^Lu]Lu-DOTA-TATE therapy of somatostatin receptor (SSTR)-2+ on patients with inoperable/metastatic PPGLs and evaluated the abnormalities in the immediate post-treatment period. This study indicates that [^177^Lu]Lu-DOTA-TATE therapy is associated with alterations in endocrine function likely from radiation exposure to SSTR2+ endocrine tissues, and these could cause clinically significant endocrinopathies. The information from this investigation should be important for the patients who receive [^177^Lu]Lu-DOTA-TATE therapy.

Furthermore, the advances of molecular mechanisms and genetics with PPGLs have led to novel therapeutic management, such as tyrosine kinase inhibitors, as reviewed by Saavedra et al. In addition, the relationship between PPGLs and other diseases has been reported. In this topic, Dai et al. reported a patient with pheochromocytoma and Langerhans cell histiocytosis (LCH), who had *EPAS1* mutation (pheochromocytoma) and *RAD54B* mutation (LCH). Wang et al. also reported a patient with paraganglioma and higher IL-6 value, who had *KIF1B* mutation. Moreover, Li et al. reported a patient with metastatic pheochromocytoma and without typical symptoms of catecholamine excess, who had *DLST* mutation. Thus, it is unclear whether the gene mutation is the cause of the phenotype/complication/co-existing disease. Future advances might explain the mechanism.

Besides, Małgorzata et al. investigated a patient with pheochromocytoma, whose ACTH and cortisol values were elevated. Previously, ACTH-producing pheochromocytomas were reported (9, 10). However, the patient described by Małgorzata et al. was negative for ACTH (and CRH). Hence, they described that catecholamine excess could activate the hypothalamic-pituitary-adrenal (HPA) axis. Considering the various complications of PPGLs, the phenotype of this case should be taken into consideration.

In conclusion, the information presented in this Research Topic provides updated perspectives of the molecular mechanisms and genetics of PPGLs and their unveiled clinicopathological implications. These enrich the perspectives of PPGLs, which could lead to improved clinical outcomes for patients with PPGLs.
